# Chronic pain in children and adolescents in Manitoba: A retrospective chart review to inform the development of a provincial service for pediatric chronic pain

**DOI:** 10.1080/24740527.2022.2094228

**Published:** 2022-08-18

**Authors:** Anna Liu, Polina Anang, Danielle Harling, Kristy Wittmeier, Kerstin Gerhold

**Affiliations:** a Department of Pediatrics and Child Health and Children’s Hospital Research Institute of Manitoba, Max Rady College of Medicine, University of Manitoba, Winnipeg, Manitoba, Canada; b Max Rady College of Medicine, Department of Psychiatry, University of Manitoba, Winnipeg, Manitoba, Canada; c Occupational Therapy, Child Health, Shared Health, Winnipeg, Manitoba, Canada; d Mississippi Center for Advanced Medicine, Madison, Mississippi, USA

**Keywords:** pediatric chronic pain, mental health, pain center, interdisciplinary health care team, opioid use

## Abstract

**Background:**

In the absence of an interdisciplinary service for pediatric chronic pain in Manitoba, pain management has been offered through a single provider outpatient setting with consultative services from physiotherapy, occupational therapy, and psychiatry since October 2015.

**Aims:**

The aim of this study was to characterize the patient population of this clinic to understand needs and inform future service development for pediatric chronic pain.

**Methods:**

Demographics and disease characteristics of all patients seen in this clinic between October 1, 2015, and February 28, 2019, were analyzed retrospectively from electronic medical records.

**Results:**

A total of 157 patients, mean age 13.1 (sd ±3.0) years, 75.2% female, with a median duration of pain of 20.5 (interquartile range [IQR] = 10.0–45.8) months at their first visit were included in the study. At baseline, 74.0% of patients experienced insomnia, 76.6% fatigue, 86.5% symptoms of anxiety, and 58.69% symptoms of depression; 80.1% showed withdrawal from physical activity, 67.1% missed school, and 10.2% reported opioid usage. Throughout their care in clinic, 83.4% of patients received physiotherapy, 17.8% occupational therapy, 49.7% mental health support, and 51.6% care from multiple services. The clinic experienced a significant increase in median referrals from 1.0 to 5.0 (IQR = 2.0–9.0) per month and wait time from 35.0 to 97.0 (IQR = 88.0–251.0) days during the observation period.

**Conclusions:**

Developing an interdisciplinary service for pediatric chronic pain will provide an opportunity to improve access, coordination, and comprehensiveness of care and to employ culturally sensitive services to improve care for children and youth living with chronic pain in Manitoba and possibly other jurisdictions with similar demographics and needs.

## RÉSUMÉ

**Contexte:** En l’absence d’un service interdisciplinaire pour la douleur chronique pédiatrique au Manitoba, la prise en charge de la douleur est proposée par un seul prestataire ambulatoire qui offre des services consultatifs de physiothérapie, d’ergothérapie et de psychiatrie depuis octobre 2015.

**Buts:** Le but de cette étude était de caractériser la population de patients de cette clinique pour comprendre les besoins et éclairer le développement futur de services pour la douleur chronique pédiatrique.

**Méthodes:** Les données démographiques et les caractéristiques de la maladie de tous les patients vus dans cette clinique entre le 1er octobre 2015 et le 28 février 2019 ont été analysées rétrospectivement à partir des dossiers médicaux électroniques.

**Résultats:** Un total de 157 patients, dont l’âge moyen était de 13,1 ans (é.-t. ±3,0) ans, 75,2 % de femmes, avec une durée de la douleur médiane de 20,5 mois (écart interquartile [IQR] = 10,0-45,8) à leur première visite étaient inclus dans l’étude. À l’inclusion, 74,0 % des patients présentaient de l’insomnie, 76,6 % de la fatigue, 86,5 % des symptômes d’anxiété et 58,69 % des symptômes de dépression ; 80,1 % montraient un retrait de l’activité physique, 67,1 % avaient manqué l’école et 10,2 % ont déclaré avoir consommé des opioïdes. Tout au long de leur traitement en clinique, 83,4 % des patients ont reçu de la physiothérapie, 17,8 % de l’ergothérapie, 49,7 % un traitement de soutien à la santé et 51,6 % des soins dispensés par de multiples services. La clinique a connu une augmentation significative des références médianes de 1,0 à 5,0 (IQR = 2,0-9,0) par mois et du temps d’attente de 35,0 à 97,0 (IQR = 88,0-251,0) pendant la période d’observation.

**Conclusions** : La mise sur pied d’un service interdisciplinaire pour la douleur chronique pédiatrique permettra d’améliorer l’accès, la coordination et l’exhaustivité des soins de même que le recours à des services adaptés à la culture pour améliorer les soins aux enfants et aux jeunes souffrant de douleur chronique au Manitoba et possiblement dans d’autres provinces et territoires ayant des caractéristiques démographiques et des besoins semblables.

## Introduction

Chronic pain in youth is common, affecting about 11% to 38% of children and adolescents.^1^ In about 5% of children, chronic pain significantly impacts multiple domains of everyday life, including sleep, mental health, physical and social activities, and schoolwork.^2^ Consultations with healthcare providers, diagnostic procedures, and medical and nonmedical treatment approaches can cause distress, interfere with daily schedules, and raise financial burden for families and the health care system before a diagnosis is eventually made and adequate treatment offered.^3^ Chronic pain in youth that is not treated effectively continues more frequently into adulthood^4^ and has recently been shown to be a risk factor for subsequent opioid misuse in adulthood.^5^ Moreover, chronic pain is well known to be an extraordinarily expensive public health problem, costing the Canadian economy roughly $40 billion in 2019.^6^

To address the complexity of chronic pain in adults and youth, interdisciplinary clinics including physicians, nurses, psychologists or psychiatrists, occupational therapists, and physiotherapists following a biopsychosocial approach are currently viewed as the standard of care.^7–9^ Beyond their effectiveness, a recent study showed a significant decrease in health care utilization and related costs over an observation period of 5 years after attending a clinic for pediatric chronic pain in Canada.^10^

The national action plan for pain in Canada^6^ aims for “people [to] have equitable and consistent access to a continuum of timely, evidence-informed, and person-centred pain care and supports across jurisdictions.” To the best of our knowledge, Manitoba remains the only Canadian province with a university-affiliated hospital without a service for pediatric chronic pain^11^ and, as such, falls short of these national recommendations.

To address the absence of an interdisciplinary pediatric pain clinic in Manitoba, a pediatric rheumatologist with expertise in chronic pain management began providing clinical care in an outpatient setting at Children’s Hospital in Winnipeg in October 2015. Collaborations were developed to include consultative services from physiotherapy, occupational therapy, and psychiatry.

The objective of this study was to characterize the patient population of this single-provider clinic to understand Manitoba’s needs for pain management and guide the planning of an interdisciplinary service to provide the standard of care to Manitoban children and adolescents living with chronic pain.

## Materials and Methods

### Study Design

A retrospective review of electronic medical records of the clinic for chronic pain in children and adolescents at Children’s Hospital of Winnipeg was conducted in 2019. The chart documents included information received by the pain clinic such as referral letters and reports from consulted health care services and information documented in the pain clinic. Ethics approval was granted by the University of Manitoba ethics board (H2019:161 (HS22796)). An informed consent waiver was accepted 90 following the Canadian Tri-Council Policy Statement “The research involves no more than minimal risk to the subjects.”

### Setting and Clinic Structure

Children’s Hospital of Winnipeg is the sole referral center for subspecialists for pediatric patients in Manitoba, Northwestern Ontario, and parts of Nunavut, Canada. The clinic for pediatric chronic pain was held one half-day a week by a single provider who by training is a pediatric rheumatologist. The clinic received referrals directly from primary care providers and pediatric subspecialists; the pain clinic provider and the second staff pediatric rheumatologist also identified patients with chronic pain who were initially referred to the rheumatology clinic to exclude a rheumatologic disease and referred them to the pain clinic. All patients referred to the clinic were triaged and seen by the pain clinic provider. Referrals for a diagnosis of complex regional pain syndrome were deemed as urgent and usually seen within 1 to 2 weeks as per recommendations of the International Association for the Study of Pain (IASP) on wait times.^12^

Nursing was available to measure youth’s vital signs in new patients. A physiotherapist (K.W.) and an occupational therapist (D.H.) were available to accept direct referrals and provide care during clinic visits on an on-call basis. For about 12 months a psychiatrist (P.A.) was available through the Consultant Liaison Service of Children’s Hospital and provided care during clinic visits. If allied and mental health services were unavailable in clinic and/or close follow-up was needed in the community, patients were referred to respective services affiliated with Children’s Hospital or to community public services or recommendations to access private community services were made. Clinic care was coordinated with community-based therapy services as possible.

During the initial clinic visit, a complete history and comprehensive physical exam were conducted. If a main diagnosis of chronic pain was not confirmed or differential diagnoses were not fully considered prior to entering the clinic, further diagnostics were initiated as appropriate. In keeping with recommended practice,^13^ patient and family education about chronic pain as a diagnosis in its own right^14^ and its biopsychosocial model was provided by the physician and allied health providers in clinic at the first and subsequent visits. Further management was based on physical, occupational, and psychological therapies. If indicated, relaxation techniques such as deep breathing were introduced by the physical therapist in clinic. If underlying physical conditions showed active disease, disease-specific treatment was optimized by the caring subspecialist. Primary care providers or subspecialists managed medications for specific pain conditions such as migraines or abdominal pain or for mental health disorders that were started prior to entry in the clinic. If indicated, pharmacological treatment for a mental health disorder was initiated or optimized after a more in-depth assessment by the psychiatrists of the Consultant Liaison Service or by the adolescent medicine specialists of Children’s Hospital in close collaboration with the pain clinic.

A treatment plan was developed together with the patient and their family during the first visit. Follow-up visits were made as required.

### Study Population

The clinic accepted referrals for patients who fulfilled the following criteria for chronic pain: (a) onset of pain before 17 years of age, (b) pain persisting >3 months, and/or (c) pain whose biological etiology could not be determined by standard biomedical assessment and imaging.^2,14^ All patients referred to the clinic from October 1, 2015, with a first visit on or prior to February 28, 2019, who fulfilled these criteria were included in the study. Following the 2016 census data of Statistics Canada,^15^ Manitoba has a population of almost 1.3 million people; 19% were under the age of 15 years, about 18% were immigrants, and about 18% of the population reported an Indigenous identity. The catchment area of the clinic encompassed large, medium, small, and rural communities, including remote communities^16^ throughout Manitoba, Northwestern Ontario, and parts of Nunavut, Canada.

### Patient Assessment

The child’s intake and follow-up history was taken by the pain clinic physician by using open-ended, nonstandardized questions. The child was preferably addressed during the interviews; the caregiver was invited to provide their perspectives as appropriate. The intake interview was comprehensive as outlined below; the interim history at follow-up visits focused on symptoms and major concerns that arose from the intake or previous follow-up visit(s) as well as on daily functioning, pain-alleviating factors, and evaluation and coordination of ongoing or further necessary therapies.

During the intake visit, the history about the child’s pain included the circumstances before and at pain onset such as acute illnesses and injuries; time of onset; pain duration and frequency; location; character; accompanying symptoms such as tingling, numbness, and light-headedness; aggravating and alleviating factors; previous diagnostics; and previous and current pharmacological, alternative, physical, and psychological therapies.

Across patients, symptoms of insomnia, fatigue, anxiety, mood disorders, and daily functioning were assessed using similarly formulated introductory questions; typical questions were as follows: (1) Sleep: “How is your sleep?” (2) Fatigue: “Do you feel tired or exhausted during the day?” (3) Anxiety: “Do you get easily nervous?”; “Do you worry about some things?” (4) Mood: “How is your mood?” (5) Daily functioning: “How is school for you?”; “Do you participate in any sport?”; “What is your favorite activity for fun?” Depending on the responses, in-depth questions followed and allowed for an open talk about sleep (duration, difficulties falling asleep or staying asleep, activities before bedtime), fatigue (including daytime napping), symptoms of anxiety (general and particular worries, history of panic attacks), mood (feeling of sadness, loss of interest, changes in appetite and weight, difficulties concentrating, suicidal thoughts), daily functioning (school attendance and performance, peer relationships, social activities with family and/or friends, physical activities and sports), and possible adverse childhood events prior to or around the onset of pain.

Patient self-reported pain scores were captured on a 21-point numeric rating scale (NRS) with intervals of 0.5, with 0 = *no pain* and 10 = *the worst pain imaginable*. Similarly, fatigue scores were captured on a 21-point NRS with 0 = *no fatigue* and 10 = *the worst fatigue imaginable*. A defined timeline for the minimum and maximum pain and fatigue scores such as “last 7 days” was not consistently used. Proxy scores were only taken for children younger than 6 years of age or for children with respective developmental delay.

Symptoms were documented in the patient’s note and summarized in the problem and diagnoses section of the note.

The social history captured also living circumstances such as household members and caregiver relationship and employment. A standardized list of conditions was used across patients to assess the medical family history; this list comprised physical, mental health, and pain disorders.

### Data Extraction and Analysis

Data were collected from electronic medical records by an independent investigator (A.L.); data and coding of 1/10 charts were validated by a second investigator (K.G.). Data extracted included referrer specialty; referral and visit dates; patient demographics; history of pain and accompanying symptoms, including symptoms of insomnia, fatigue, anxiety, and depression; and functional limitations including withdrawal from physical activity and school. Symptoms documented in the history and impression section and in the problem list of the patient’s note were compared and further validated with the physician in case of inconsistency before being coded as being present for data extraction. Medications and services used for disease management prior to and during clinic enrollment and past medical and family history were recorded.

Data were entered into REDCap^17^ and analyzed using the Statistical Package for the Social Sciences v25.^18^ Descriptive measures applied depended on the data distribution. Kruskal-Wallis analysis was used to compare referral frequency and wait time over the observation period from 2015 to 2019; the significance level was set with α = 0.05.

Data for patients who did not attend the clinic were only included in referral frequency and wait time analyses. To avoid generating artificially low wait times, patients referred by the pain clinic provider out of her rheumatology clinic were excluded in the wait time analysis, because she covered the first clinic visit in her rheumatology clinic and booked the patient as a follow-up visit into the pain clinic. To be transparent, we calculated the wait time between the referral and the first originally booked appointment as well as the wait time between the referral and the actual first visit a patient attended. Wait times between the referral and the originally scheduled appointment versus the actual first visit differed if a patient (a) did not attend their originally scheduled first appointment but was rebooked after a follow-up call by the clinic, (b) had called in advance and rebooked their first appointment, or (c) was rescheduled on the initiative of the clinic.

## Results

### Study Population

A total of 181 patient charts were screened; 13 were excluded because the time of the referral was outside the study period and 2 because their diagnosis did not meet the inclusion criteria. Of 166 patients triaged and booked into the clinic, 9 patients did not attend the clinic at any time. These 9 patients had a mean age of 12.3 years old (SD ±3.3); 6 were females; they were referred from five specialties. Six of these patients were referred for management of musculoskeletal pain, 1 for abdominal pain, and 2 for pain in several locations. Four patients were from large communities, and 4 patients were from small and rural communities; 1 patient could not be located. A total of 157 patients attended the clinic between October 1, 2015, and February 28, 2019.

### Referral Frequency and Wait Times

The number of referrals to the clinic increased significantly during the observation period with a median of 1.0 per month in 2015, 2.5 (interquartile range [IQR] = 2.0–4.0) per month in 2016, 5.5 (IQR = 2.8–7.8) per month in 2017, and 5.0 (IQR = 2.0–9.0) per month in 2018 (*P* = 0.036).

Of the 166 triaged patients, 67.5% were referred by pediatric subspecialists affiliated with Children’s Hospital (Table 1). Thirty-seven patients were referred by the clinic provider from her rheumatology clinic; these patients were scheduled for a follow-up visit in the pain clinic and excluded from the wait time analysis. The average wait time from the day of referral (referral date) to the originally scheduled date of the first appointment was a median of 56.0 (IQR = 35.0–88.5) days and was similar to the wait time until the actual first visit of 57.0 (IQR = 31.0–92.0) days. Thirty-two first visits were rescheduled by the clinic; 21 visits were scheduled at an earlier than the originally scheduled appointment date, and 11 visits were scheduled later than the initially booked appointment date. Twenty-six patients canceled and four patients did not attend their originally booked appointment; these patients were rebooked at the next available time. For ten patients it is unknown whether the first appointment was rescheduled by the clinic or patient. The wait time between the referral date and the actual first visit significantly increased over the observation period with a median of 35.0 days for patients seen in 2015, 46.0 (IQR = 27.5–63.8) in 2016, 57.0 (IQR = 27.8–85.5) in 2017, 57.0 (IQR = 31.5–110.0) in 2018, and 97.0 (IQR = 88.0–251.0; *n* = 7) days for patients seen in 2019 (*P* = 0.012). The increase in wait time between the referral date and the initially scheduled first appointment was similar, from 35.0 days in 2015 to 88.5 (IQR = 80.5–100.3) days in 2019 (*P* = 0.008).

**Table 1.** Referrer specialties.

Referrer to pain clinic (*N* = 166)	*n* (%)
General pediatrics, community-based	21 (12.7)
Pediatric rheumatology Provider at the pain clinic Non-pain specialist	37 (22.3) 30 (18.1)
Pediatric gastroenterology	21 (12.7)
Pediatric neurology	12 (7.2)
Sports medicine	8 (4.8)
Orthopedics	7 (4.2)
Pediatric emergency	6 (3.6)
Pediatric inpatient service	6 (3.6)
Pediatric hematology oncology	4 (2.4)
Pediatric rehabilitation services	2 (1.2)
Genetics	2 (1.2)
Other	10 (6.0)

A total of 166 referrals to the pain clinic were received between October 1, 2015, and December 31, 2018, with a first patient visit booked on or prior to February 28, 2019. Most pediatric subspecialists who referred to the pain clinic were affiliated with Children’s Hospital.

### Demographics

The mean age of the 157 patients seen in the clinic was 13.1 (SD ±3.0) years; 75.2% were female (Table 2). More than one third (38.2%) of patients were living in small or rural/remote communities. Almost two thirds (58.6%) of patients identified as being of Canadian/Europeans ancestry (Table 2).

**Table 2.** Patient demographics and disease characteristics at baseline.

**Female**, *n* (%) (*N* = 157)	118 (75.2)
**Age at first visit**, *n* (%) (*N* = 157)	
0–4 years	2 (1.3)
5–9 years	21 (13.4)
10–14 years	69 (43.9)
15–17 years	65 (41.4)
**Ethnicity (self-identified)**, *n* (%) (*N* = 128)	
Caucasian	75 (58.6)
Indigenous	11 (8.6)
Asian	4 (3.1)
Other^a^	37 (28.9)
**Living area**, *n* (%) (*N* = 157)^b^	
Large communities (population >100,000)	89 (56.7)
Medium communities (population 30,000–99,000)	8 (5.1)
*S*mall communities (population 1000–29,999)	40 (25.5)
Rural communities (population <1000 or <400/km^2^)	30 (19.1)
**Duration of pain**, median [months] (IQR) (*N* = 124)	20.5 (10–45.8)
Female (*n* = 96)	19 (9.25–40.5)
Male (*n* = 28)	24 (10.5–69.0)^c^
**Pain intensity, 21-point NRS**, median (IQR)	
Pain at first visit (*N* = 114)	4.8 (2.4–6.0)
Pain at first visit, female (*n* = 89)	5.0 (2.3–6.0)
Pain at first visit, male (*n* = 25)	4.0 (2.3–6.0)
Minimum pain (*N* = 62)	1.3 (0.0–3.3)
Maximum pain (*N* = 77)	8.7 (8.3–10.0)
**Fatigue intensity, 21-point NRS**, median (IQR) (*N* = 91)	4.0 (0.0–6.0)
Female (*n* = 69)	4.5 (0.0–6.5)
Male (*n* = 22)	2.3 (0.0–5.0)^d^
**Accompanying symptoms**, *n* (%)	
Disordered sleep (*N* = 131)	97 (74.0)
Fatigue (*N* = 111)	87 (76.6)
Symptoms of anxiety (*N* = 96)	83 (86.5)
Symptoms of depression (*N* = 87)	51 (58.6)
**Functional impairment**, *n* (%)	
Withdrawal from physical activity (*N* = 136)	109 (80.1)
Withdrawal from gym class (*N* = 87)	55 (63.2)
– Partial withdrawal	25 (28.7)
– Full withdrawal	30 (34.5)
Missing school (*N* = 140)	94 (67.1)

Percentages presented in this table may not equal 100% due to rounding.

^a^Other: Parents identified as having more than one ethnic background, including Indigenous (Inuit, Métis, Status First Nations, non-status First Nations), European, Caribbean, Latin, Central and South American, African, Asian, and Oceania origins.^15^

^b^Ten patients (6.4%) were from small (*n* = 7) and rural (*n* = 3) communities in Northwestern Ontario.

The difference in the duration of pain and the fatigue intensity scores between males and females at baseline was statistically significant: ^c^*P* = 0.04; ^d^*P* = 0.05.

### Disease Characteristics at Baseline

Patients were experiencing pain for a median of 20.5 (IQR = 10.0–45.8) months at baseline (Table 2), with males experiencing pain about 5 months longer than females. Pain intensity scores did not differ significantly between females and males, but fatigue intensity scores were higher in females than in males at baseline.

Musculoskeletal pain was the most common main pain type (72.6%), followed by abdominal pain (17.1%), headaches (8.9%), chest pain (0.6%), and pain in other locations (0.6%); patients experienced pain in a median of 2.0 (IQR = 1.5–3.0) different locations. Accompanying symptoms of insomnia, fatigue, anxiety, and/or depression were reported by 84.7% of patients during their first clinc visit; 84.0% of patients experienced at least one domain of functional impairment (Table 2).

### Past Medical and Family History and Exposure to Psychosocial Stressors

Thirty-four percent of patients reported ongoing physical health concerns. Seven patients (4.5%) reported acute illnesses that had resolved by their first visit. Thirty-four patients (21.7%) were noted in the referral letter to have at least one mental health diagnosis or reported such a diagnosis during their intake visit; among them, 17 patients (10.8%) were documented to have an anxiety disorder, 10 patients (6.4%) a depressive disorder, 5 patients (3.2%) attention deficit hyperactivity disorder, and 12 patients (7.6%) self-harm behavior and/or a previous suicide attempt. One-third of patients reported potential psychosocial stressors at their first visit (Table 3); however, not all patients vocalized them as a source of stress. Eighty patients (52.6%) had a first-degree relative with a mental health disorder or chronic pain; in 5 patients the family history was unknown (Table 3).

**Table 3.** Individual factors of the biopsychosocial model of pain (*N* = 157).

Patient-reported past medical history and potential psychosocial stressors			
Medical conditions	*n* (%)	Potential psychosocial stressors	*n* (%)
Chronic constipation	6 (3.8)	Parental separation	29 (18.5)
MSK injury	6 (3.8)	Bullying/stigmatization^a^	17 (10.8)
Concussion	5 (3.2)	Illness or death of loved one	10 (6.4)
Arthritis	5 (3.2)	Adoption^b^	6 (3.8)
Other	39 (24.8)	Other	7 (4.5)
More than one medical conditions	8 (5.1)	More than one potential stressor	14 (8.9)
Total number of patients	53 (33.8)	Total number of patients	55 (35.0)
History of patients’ first-degree family members			
Mental health	*n* (%)	Chronic pain	*n* (%)
Anxiety	22 (14.0)	Headaches	15 (9.6)
Depression	20 (12.7)	Joint pain	14 (8.9)
ADHD	12 (7.6)	Fibromyalgia	12 (7.6)
Drug and/or alcohol use disorder	5 (3.2)	Back pain	11 (7.0)
Other	18 (11.5)	Chronic abdominal pain	5 (3.2)
More than one mental health concern	32 (20.4)	Other	7 (4.5)
		More than one chronic pain diagnosis	9 (5.7)
Total number of patients (%)	45 (28.7)	Total number of patients (%)	55 (35.0)

Medical conditions diagnosed prior to the first pain clinic visit, psychosocial stressors, and exposure to mental health concerns and chronic pain of first-degree relatives are presented because they may contribute to pain perception and chronic pain development following the biopsychosocial model of illness.^13^ Potential psychosocial stressors were identified in discussion with patients at their first visit. However, not all patients vocalized them as a source of stress.

^a^Bullying is not clearly defined because it was self-reported; it may encompass stigmatization and criticism in relation to chronic pain.

^b^Adoption is listed because it has been described to be associated with prenatal or postnatal/preadoption stress for the child or stress during the adoption transition.^17^

MSK = musculoskeletal; ADHD = attention deficit hyperactivity disorder.

### Medications at Baseline

Prior to their first clinic visit, 143 (91.1%) patients reported usage of at least one medication. Among them, 100 (69.9%) patients reported use of nonsteroidal anti-inflammatory drugs, 51 (35.7%) acetaminophen, 31 (21.7%) antidepressants, 16 (11.2%) opioids (10.2% of all patients), and 8 (5.6%) gabapentin; in addition, 5 (3.5%) patients reported use of medical cannabis.

### Treatment by Allied Health Services

**Figure 1.**
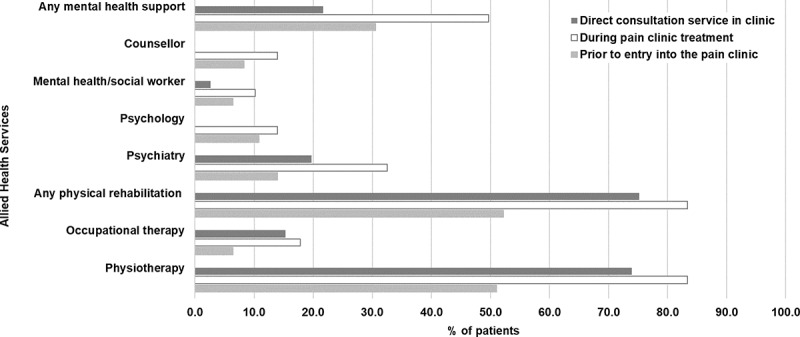
Allied health services: Proportion of patients receiving allied health services prior to entry and during treatment in the chronic pain clinic (*N* = 157). Patients who had not received allied health services were either referred to a particular service during their treatment period at the pain clinic or a service was recommended and the family was going to explore options in the community by themselves. Restricted consultative services from physiotherapy, from occupational therapy, and for one year from the Psychiatry Liaison Service of Children’s Hospital were available to the pain clinic, and a proportion of patients received these services during their clinic visits and/or independently from the clinic visits in an outpatient setting.

Prior to their first visit, 23.6% of patients received treatment by more than one allied health service. During their period of care in our clinic, 83.4% of patients received physiotherapy, 17.8% occupational therapy, and 49.7% mental health support (Figure 1); 52.2% received treatment by more than one service. Fourteen percent of patients were not involved in physical rehabilitation services, and 41.4% did not receive mental health support within the observation period of the study.

### Follow-up and Last Visits

**Figure 2.**
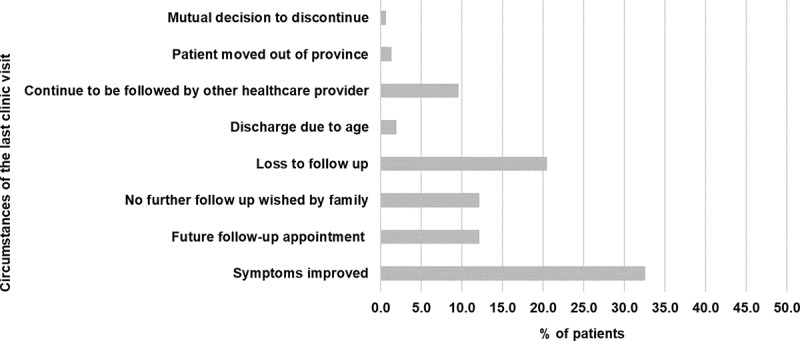
Reasons for the last visit in the pain clinic (*N* = 142). Documentation was missed in 15 charts (9.6%).

Patients had a median of 2.0 (IQR = 2.0–3.0) visits in the pain clinic; 33 patients (21.0%) were seen only once, and 11 were lost to follow-up. Improvement in symptoms was documented in about one-third of patients at discharge (Figure 2).

## Discussion

In the absence of an interdisciplinary pediatric pain clinic in Manitoba, we aimed to characterize the patient population of our single-provider pain clinic to understand the needs and inform the planning of an interdisciplinary provincial service to provide the standard of care to Manitoban children and adolescents living with chronic pain.

IASP, the Canadian Pain Society, and the national action plan for pain in Canada, endorse the need for interdisciplinary clinics to treat chronic pain in adults and children using a functional rehabilitation approach.^6–9,14^ Those clinics have been shown to be effective in lowering pain, improving function including school attendance, and being cost-effective.^7,10,20^ Widely discussed barriers of implementing such a service include a lack of clearly defined admission criteria and distinct treatment approaches particularly for children’s pain, limited resources, competing demands, and payment structures.^21^ Similar to other international jurisdictions, the information from this study may aid in understanding our province’s unique needs and help to guide advocacy and planning for a provincial interdisciplinary service for chronic pain in children and adolescents.^22^

Comparable to other regions,^8,22^ most patients with chronic pain referred to our clinic were adolescent females experiencing pain for over a year before entering the clinic, as well as mental health concerns, functional impairment, and exposure to potential psychosocial stressors. Further research on the longer symptom duration prior to treatment in males in our cohort will need to consider sex and gender differences in pain perception, illness behavior, and cultural and social viewpoints of patients and providers.^23^

That more patients cited musculoskeletal pain as the main pain type compared to headaches and abdominal pain in other centers^8^ is likely related to a pediatric rheumatologist running the clinic. A greater awareness of the pain clinic within the province’s pediatric tertiary care facility may explain why most patients were referred by subspecialists working in the same facility. Practice and referral patterns of these subspecialists likely affected characteristics of the patient population referred to the pain clinic.

In our cohort, 91.1% of patients reported the use of at least one medication at their baseline visit, among whom 11.2% reported the use of opioids. A very recent population-based study on management of chronic non-cancer musculoskeletal pain showed that opioids were rarely prescribed for children and adolescents under the age of 18 years but in 22.9% of visits of young adults of over 18 years of age.^24^ The significant opioid usage in our pediatric clinic population may reflect the burden of pain and the lack of resources for pain management. A recently found decline in opioid prescriptions in adult patients with fibromyalgia was attributed to opioid monitoring programs, rising awareness of the addictive and harmful effects of opioids, and the growing evidence of nonopioid therapies that are effective in treatment of chronic pain.^25^ However, functional rehabilitation programs have been shown to reduce opioid usage in patients who were taking opioids when they entered such a program.^26^ Whether a functional rehabilitation approach through an interdisciplinary pain service will help reduce opioid usage in our children and adolescents experiencing chronic pain will need to be evaluated.

About 15% to 25% of Canadians experience mental illness prior to the age of 19 years.^15^ The coexistence of anxiety in younger children and depression in older children/adolescents with chronic pain is well described in the literature.^27,28^ Mental illness in youth with chronic pain is more likely associated with functional impairment, such as limited school attendance.^5^ In our clinic, 84.0% of patients presented with symptoms of insomnia, fatigue, anxiety, and/or depression; a formal mental health diagnosis was not made without involvement of psychology or psychiatry services. Almost half of our patients (48.4%) did not receive mental health support at any time prior to or during their clinic enrollment; unfortunately, we did not document the proportion of patients on a wait list or denied enrollment into the service. The relatively high proportion of 32.5% of patients seen by psychiatry was related to the availability of our colleague from the Consultant Liaison Service during clinics and the overall shorter wait times to psychiatry compared to psychology services.

Treatment challenges without mental health and rehabilitation services consistently available in clinic included additional wait times for patients referred to these services (e.g., wait times to see a clinical psychologist covered by the public health care system were up to 12 months with the proportion of psychologists far below the national average: 19 versus 49/100,000 in 2017^29^). Though our pain service functioned to support timely communication between providers (including mental health, allied health, primary treating physician, and subspecialists), the constraints of a half-day clinic with services provided through consultation lessened the chances for consistent messaging and integrated planning with families. Reflecting these challenges, our clinic discharged only about a third of its patients due to improvement of symptoms; however, about a quarter of patients continued therapy beyond the study period.

Physical and mental health concerns in first-degree relatives of pediatric patients with chronic pain may impact children’s pain perceptions and daily functioning.^30^ The frequent co-occurrence of mental health illnesses or chronic pain experiences in family members in our cohort warrants consideration of integrating family therapy when planning an interdisciplinary clinic.

More than one-third (38.2%) of our patients were living in small, rural, and remote communities. Further, even though a small number in total, four of nine patients who never showed to our clinic were from small and rural communities. Small, rural, and remote communities in Manitoba may have limited access to health care services such as physical therapy and mental health services; visiting health care facilities providing these services may be difficult due to availability of transportation and/or harsh weather conditions. A particular challenge for a provincial pediatric pain center will be service coverage of these areas. Outreach clinics, virtual care, and the Extension for Community Healthcare Outcomes model to educate frontline health care providers in pain management have been developed and may improve access, outcomes, and service use of a pediatric pain center with its main location in the capital.^31–33^

Patient demographics differed from the Manitoba population particularly concerning its Indigenous population. About 18% of the Manitoba population are Indigenous peoples.^15^ Our clinic population of 8.6% Indigenous peoples may reflect the fact that the clinic was relatively unknown to community providers. This discrepancy may also reflect the multiple barriers to accessing health care for Indigenous peoples that exist because of systemic racism and colonialism^34^ rather than a lesser pain prevalence.^35^ Cultural competence, cultural relevance, safety, and equity in access to care must be an integral part of a future interdisciplinary pediatric pain clinic in Manitoba.

The estimated median wait time for a first appointment in publicly funded adult and pediatric pain centers in Canada is 6 months.^8^ The median wait time for a new assessment in pediatric pain clinics in Australia has been estimated with 32.5 days.^36^ The wait time in our clinic was increasing from 35.0 days (or 1.2 months) in 2015 to 97.0 days (or 3.2 months) for patients seen in 2019. The relatively lower wait time compared to the current average of Canadian pain clinics was likely related to the clinic being relatively unknown. The IASP-recommended wait time is 8 weeks at the most.^12^ The wait time experienced by families in this cohort has been above this indicator since 2017. The increase in referrals and subsequently wait time likely demonstrates an increased awareness of the clinic and mirrors the demand of such a service in Manitoba with its total population of about 300,000 children up to the age of 17 years.^16^ The increasing demand must be considered when planning clinic hours and staffing for a provincial center.

In 2006, the Initiative on Methods, Measurement, and Pain Assessment in Clinical Trials identified eight core outcome domains for clinical trials and registries in pediatric chronic pain, including pain intensity, physical functioning, symptoms/adverse events, global satisfaction with treatment, emotional functioning, role functioning, sleep, and economic factors.^37^ A review on implementation of these domains in recent research showed that only the domain of pain intensity was consistently used as outcome measure.^38^ Due to the limited uptake of these suggested domains, a revised core set of outcomes for clinical trials in pediatric chronic pain clinics was suggested very recently.^39^ The mandatory domains of the core set included pain severity, pain interference with daily living, overall well-being, and adverse events; optional domains were emotional functioning, physical functioning, and sleep. During intake visits, the content of these domains was captured in our clinic by assessing pain intensity and limitations due to pain, social and mental well-being, sleep and fatigue, daily functioning, and psychosocial stress factors including childhood adverse events. However, we used nonstandardized, open-ended questions, and follow-up visits included these areas only as indicated from the intake visit or previous follow-up visits or if new concerns were voiced by the patient or caregiver. Therefore, one major limitation of our study is the retrospective nature with visits being not standardized. Consequently, questions were not consistently asked the same way or asked at all to every patient; resulting missing values may have limited the study’s internal validity. To address the lack of standardized outcome measures in our clinic, we envision incorporating validated patient reported outcomes of the pan-Canadian pediatric pain registry as key initiative to inform the next iteration of our program.^40^ In conclusion, our data suggest a demand for a service for children and adolescents living with chronic pain throughout the province of Manitoba and its catchment area of neighboring provinces. Such a service will ideally comprise a team of dedicated physicians, nurses, physiotherapists, occupational therapists, psychologists or psychiatrists, and social worker with expertise in family therapy available for all patients entering the clinic. Developing a pediatric clinic for chronic pain following such an interdisciplinary, rehabilitative model will provide an opportunity to improve coordination and comprehensiveness of care, to implement culturally sensitive services, to increase access to care in rural and remote communities, to aim for suitable wait times to improve care for children and adolescents living with chronic pain, and to reduce costs related to health care utilization in pediatric chronic pain.^19^ Whether such a clinic will reduce the transition of pediatric to adult chronic pain or opioid use in adulthood remains to be seen.
